# COVID-19 Vaccine Hesitancy in Italy: Predictors of Acceptance, Fence Sitting and Refusal of the COVID-19 Vaccination

**DOI:** 10.3389/fpubh.2022.873098

**Published:** 2022-04-29

**Authors:** Cristina Zarbo, Valentina Candini, Clarissa Ferrari, Miriam d'Addazio, Gemma Calamandrei, Fabrizio Starace, Marta Caserotti, Teresa Gavaruzzi, Lorella Lotto, Alessandra Tasso, Manuel Zamparini, Giovanni de Girolamo

**Affiliations:** ^1^Unit of Epidemiological Psychiatry and Evaluation, IRCCS Istituto Centro San Giovanni di Dio Fatebenefratelli, Brescia, Italy; ^2^Service of Statistics, IRCCS Istituto Centro San Giovanni di Dio Fatebenefratelli, Brescia, Italy; ^3^Centre for Behavioral Science and Mental Health, Istituto Superiore di Sanità, Roma, Italy; ^4^Department of Mental Health and Drug Abuse, Azienda Unitá Sanitaria Locale (AUSL) Modena, Modena, Italy; ^5^Department of Developmental Psychology and Socialization, University of Padova, Padova, Italy; ^6^Department of Humanities, University of Ferrara, Ferrara, Italy

**Keywords:** trust, conspiracy, vaccination, perceived risk, restrictions, protective behaviors

## Abstract

**Background:**

The hesitancy in taking the COVID-19 vaccine is a global challenge. The need to identify predictors of COVID-19 vaccine reluctance is critical. Our objectives were to evaluate sociodemographic, psychological, and behavioral factors, as well as attitudes and beliefs that influence COVID-19 vaccination hesitancy in the general population of Italy.

**Methods:**

A total of 2,015 people were assessed in two waves (March, April and May, 2021). Participants were divided into three groups: (1) individuals who accepted the vaccination (“accepters”); (2) individuals who refused the vaccination (“rejecters”); and (3) individuals who were uncertain about their attitudes toward the vaccination (“fence sitters”). Group comparisons were performed using ANOVA, the Kruskal-Wallis test and chi-square tests. The strength of the association between the groups and the participants' characteristics was analyzed using a series of multinomial logistic regression models with bootstrap internal validation (one for each factor).

**Results:**

The “fence sitters” group, when compared to the others, included individuals of younger age, lower educational level, and worsening economic situation in the previous 3 months. After controlling for sociodemographic factors, the following features emerged as the main risk factors for being “fence sitters” (compared with vaccine “accepters”): reporting lower levels of protective behaviors, trust in institutions and informational sources, frequency of use of informational sources, agreement with restrictions and higher conspirative mentality. Higher levels of COVID-19 perceived risk, trust in institutions and informational sources, frequency of use of informational sources, agreement with restrictions and protective behaviors were associated with a higher likelihood of becoming “fence sitters” rather than vaccine “rejecters.”

**Conclusions:**

The “fence sitters” profile revealed by this study is intriguing and should be the focus of public programmes aimed at improving adherence to the COVID-19 vaccination campaign.

## Introduction

The coronavirus disease 2019 (COVID-19) pandemic has caused havoc in global healthcare systems and has had a significant impact on different aspects of daily life (1–3), prompting pharmaceutical companies to urgently create vaccines and monoclonal antibodies to combat this public health emergency. The development of safe and effective COVID-19 vaccinations is widely regarded as the first step toward a long-term solution to the pandemic. Indeed, a high vaccination rate would ensure the pandemic's eradication or control. However, as the pandemic has progressed, the number of people willing to get vaccinated has declined (4). Even before the COVID-19 crisis, the World Health Organization (WHO) confirmed vaccine hesitancy as one of the top 10 global health threats for 2019. The SAGE Working Group has defined vaccine hesitancy as “*a delay in acceptance or refusal of vaccines despite availability of vaccination services*” adding that “*vaccine hesitancy is complex and context specific, varying across time, place and vaccines*” (5, 6).

Vaccine hesitancy is influenced by factors such as confidence (do not trust vaccine or provider), complacency (do not recognize a need for a vaccine, do not value vaccination) and convenience (accessibility to vaccines) (7). COVID-19 vaccine hesitancy has been frequently linked to fears that the vaccinations are unsafe, they were developed too quickly, they may induce adverse effects (e.g., infertility, death), they are pointless due to COVID-19's innocuous nature, and they are designed to inject microchips (8–13). Moreover, some conspirative theories suggest that pharmaceutical corporations produced and disseminated the virus in order to sell their medications and vaccines (14, 15).

Therefore, it is critical to identify the predictors of COVID-19 vaccine hesitancy so that specific *ad hoc* public programmes and communication strategies can be implemented to inform governments, increase the positive responses to the COVID-19 vaccination campaign (including the “booster dose” or periodic), and establish guidelines for better managing future pandemics. Previous studies have indicated that the factors affecting vaccine intention and uptake differ substantially depending on the country, culture and socioeconomic conditions. COVID-19 vaccine hesitancy has been associated with younger age (16–21), female gender (12, 16, 18, 19, 22–24), adherence to conspiracy theories (14, 16, 18), belief that the risks related to the COVID-19 pandemic had been exaggerated by the media and that the pandemic would not last much longer (25), low perceived risk (16, 18, 24, 26), lower use of traditional and authoritative information sources (27), poor perception of government measures (20) and low trust/confidence in scientists, healthcare workers, health systems and government (12, 16, 20, 22, 28). Furthermore, a recent Italian study (29) focusing on vaccination hesitancy in case people will be tested positive for COVID-19 (i.e., post-positive reluctance) and those who relied on others to get vaccinated (i.e., free-riding intention) discovered that these two groups had a medium or high frequency of media information use and medium or high levels of conspiracy-mindedness. Various studies have revealed contrasting results for income and education. Specifically, some studies found that vaccine reluctance was associated with lower education (16, 18, 30) and lower income (16, 20, 30), while others discovered that vaccine hesitancy was higher in people with a university/postgraduate education degree (22), college-level education (26) or higher monthly income (12).

Despite their importance, most of these studies have focused on attitudes and intentions toward vaccines, rather than on behavior (acceptance or refusal), mostly when they were not available yet (i.e., until the end of 2020). Furthermore, limited studies have investigated the predictors of COVID-19 vaccine hesitancy in the general Italian population (31–35), and only a few study have looked into the predictors that differentiate individuals who accepted the vaccination (“accepters”), individuals who refused the vaccination (“rejecters”) and individuals who were uncertain about getting vaccinated when the vaccine will be available for them (“fence sitters”).

Therefore, the present study aimed to determine which sociodemographic, psychological, belief and behavioral factors influence COVID-19 vaccine hesitancy in a representative sample of the Italian general population, with a special focus on “fence sitters” profiles. According to Verger and Dubé (36), “fence sitters” are a primary target for measures aimed at increasing vaccination coverage. In particular, we aimed at: (1) exploring sociodemographic, psychological, belief and behavioral differences between “accepters,” “rejecters” and “fence sitters,” and (2) identifying the factors that most predict the likelihood of being “accepters” vs. being “fence sitters,” and the likelihood of being “rejecters” vs. being “fence sitters.”

## Methods

### Participants and Procedures

**Figure 1:**
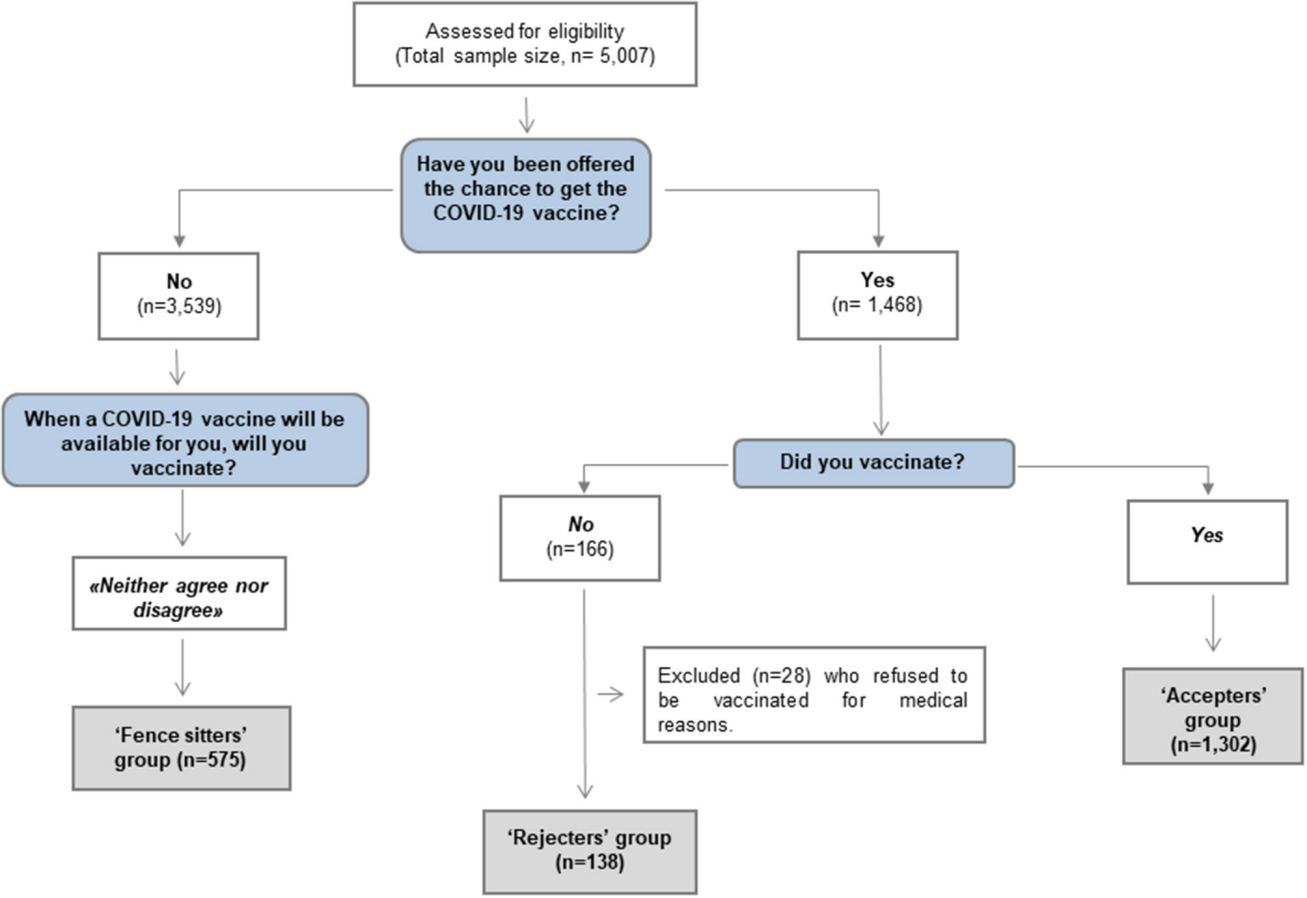
Flow-Chart of groups' stratification.

This cross-sectional study is part of a larger project promoted by the WHO Regional Office for Europe called “*Monitoring knowledge, risk perceptions, preventive behavior and trust to inform pandemic outbreak response*” and conducted in 33 countries (see WHO 2021 for the full protocol). The Italian survey *COVID Monitoring in Italy* (“COMIT”) (registered ISRCTN on 11/05/2021, ID: ISRCTN 26200758) was conducted in four waves (January–May 2021) with a sample of 10,013 people aged 18–70 years old using an online questionnaire designed *ad hoc* by WHO. In this manuscript, we will discuss specific data on behavior and attitudes toward the COVID-19 vaccine, involving 2,015 participants from the Italian general population and collected in the last two waves (when vaccines become accessible to a large portion of the population): Wave 3 (23^rd^ March-2^nd^ April 2021) and Wave 4 (7^th^-20^th^ May 2021). Figure 1 shows the flowchart for sampling selection.

A detailed sampling plan was designed to obtain a representative stratification of the Italian adult population. The following variables were employed to stratify the participants: by gender, by age (18–34, 35–44, 45–54, and 55–70 years); geographical area (Northwest, Northeast, Center, South, and Islands), size of living centers (above and below 100,000 inhabitants), education level (up to lower middle school, beyond lower middle school) and employment (employed, not employed). According to the most recent data from the Italian Statistics Institute (ISTAT, 12/31/2019), a weighting technique was conducted at the end of each wave to precisely restore the proportionality of the total sample investigated with the reference population. The main socio-demographic and geographic variables were weighted (e.g., sex by age by geographical area, occupation, education, geographical area and size of living centers). The survey study was conducted by Doxa S.p.A. and carried out using an online panel utilizing the computer-aided web interview technique (CAWI) and the Confirmit software platform. All participants, as a representative sample of the target population, received an invitation by e-mail to fill the online interview via a link: first, informed consent was requested and then the questionnaire was accessed. The average administration time was ~20 min. This study was approved by the Ethics Committee and all participants gave their informed consent.

### Measures

The WHO questionnaire covered 21 different subject categories, including knowledge, risk perception, preventive behaviors, and trust. Following the WHO's translation guidelines, the questionnaire was translated into Italian. Forward translation, panel experts, back-translation, pre-test and cognitive interviews and development of the final edition were all part of the process. In this article, we considered the following domains of the WHO questionnaire: socio-demographic characteristics (i.e., age, sex, education level, occupational status and financial situation), personal direct and indirect experience with COVID-19, COVID-19 perceived risk, trust in healthcare institutions, trust in information provided by media, trust in information provided by institutions, frequency of use of media information sources, frequency of use of health information sources, agreement with restrictions enforced by the Italian government during the pandemic, conspiracy mentality assessed using the Conspiracy Mentality Questionnaire (CMQ) (37), wellbeing assessed through the WHO-5 (38) and three items of the Brief Resilience Scale (39). Detailed information on the items covered in each factor is presented in Supplementary Table S1.

The willingness to be vaccinated was evaluated using three questionnaire items (see Figure 1). The “rejecters” group was represented by individuals who refused the COVID-19 vaccine (with the exception of those who were unable to get the vaccination because of medical reasons); the “accepters” group included those who accepted the vaccine; finally, the “fence sitters” group included those who had not been offered the vaccine at the time of the survey and who chose the middle point “*neither agree nor disagree”* on the Likert 7-point scale at the item exploring their willingness to get vaccinated in the near future.

Since the three groups matched distinct demographic strata in terms of vaccination time schedules at the time of the survey, *ad-hoc* methodological changes were made as needed (see next section). These adjustments were required due to differing vaccination access: in fact, “accepters” and “rejecters” belonged to a subgroup of the population (e.g., older people, health workers, educational staff and individuals with chronic diseases) who were offered the vaccination first, whereas “fence sitters” belonged to a larger stratum of the general population who were excluded from the initial vaccination schedule and had to wait longer to receive the vaccine as per the government policy.

### Statistical Analyses

Descriptive statistics consisted of means and standard deviations (SD) for continuous variables and frequency tables for categorical variables. The Kolmogorov-Smirnov and Shapiro-Wilk tests were utilized to analyse whether continuous variables were normally distributed. ANOVA (or the related non-parametric Kruskal-Wallis test if the investigated variable was not normally distributed) was used to compare groups in terms of mean scores, and multiple comparisons were adjusted with Bonferroni *post-hoc* technique. The relationships between categorical variables and groups were examined using the chi-square test.

Due to the large number of WHO items, a data reduction approach based on exploratory factor analysis was applied to derive a few key factors (see Supplementary Table S1). To assess the strength of the association (expressed in terms of Odds Ratio and Nagelkerke's R^2^ [N-R^2^] index) between the study groups and the subjects' features, a series of multinomial logistic regression models (one for each factor) were employed with groups (“accepters,” “fence sitters” and “rejecters”) as dependent variables and behavioral factors as independent variables. To account for possible biases due to the different subpopulations in the three groups, we included the main findings of the descriptive analyses related to these three groups in the multinomial logistic regression model, and the models were adjusted for age, gender, chronic disease, educational level, working (and health-working) status, economic situation in the last 3 months and COVID-19 infection, to manage the potential confounding effect caused by the disparity between the two groups who were offered the vaccination (“accepters” and “rejecters”) and the group that was not yet offered the vaccination (“fence sitters”) and was assessed on their willingness to get vaccinated in the future. The results were confirmed using the bootstrap method on 500 bootstrap samples to account for the imbalance of the three groups (40). Analyses were performed using R (41) and SPSS version 27.0.

## Results

**Figure 2:**
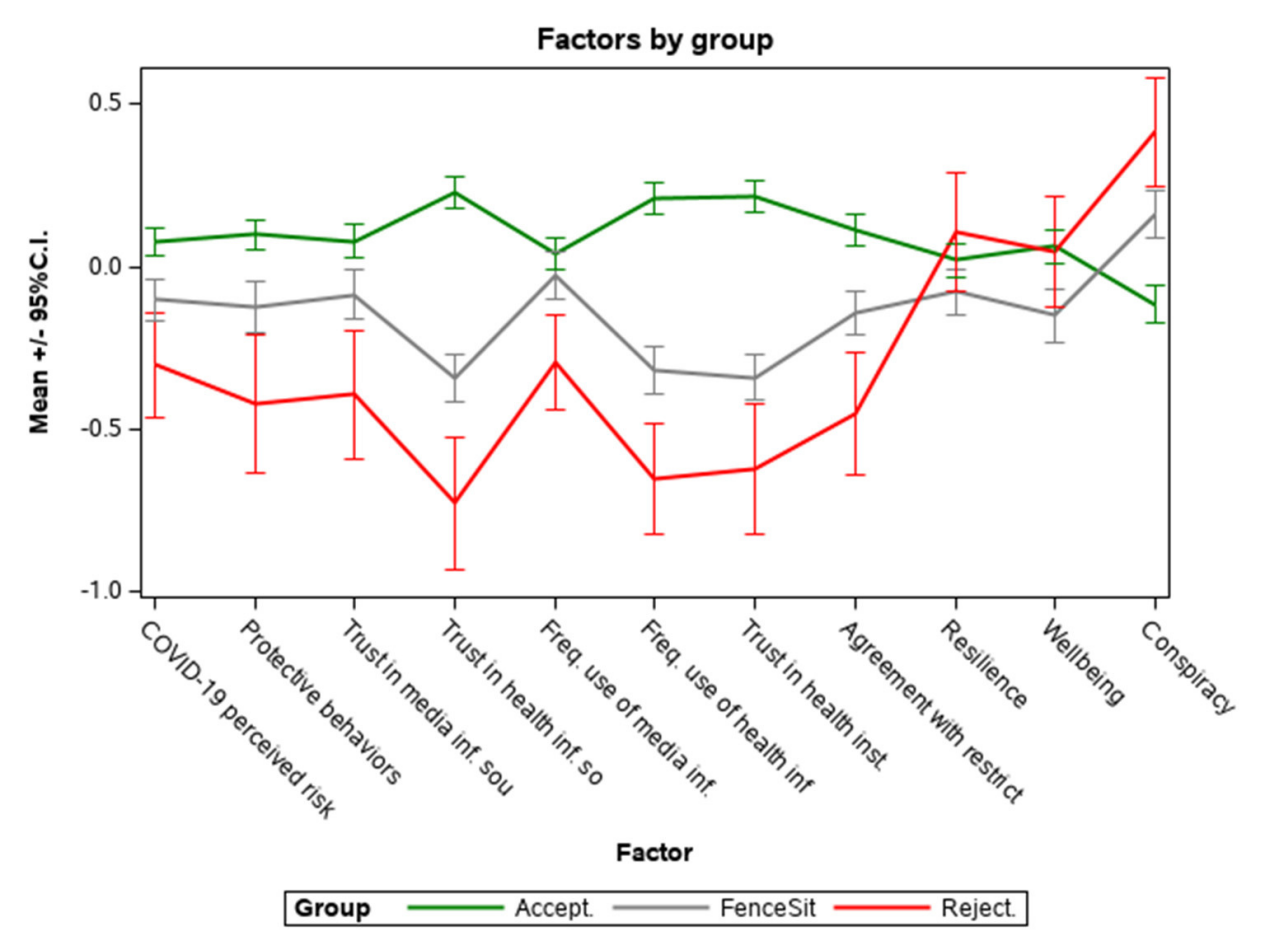
Average scores of factors in the three groups (Accepters, Fence Sitters, and Rejecters)*. *To facilitate visualization and interpretation, Wellbeing and Conspiracy scores were standardized. Error bars represent 95% confidence intervals.

Table 1 and Figure 2 show the sociodemographic, psychological, belief and behavioral characteristics and differences between the three subgroups. As expected, almost all variables were distributed differently across the three groups. In terms of socio-demographic features, “fence sitters” were younger (M_Age_ = 43.1, SD = 11.9) than “accepters” or “rejecters” (M_Age_ = 50.5 and 49.9, SD = 11.8 and 11.9, respectively) (*p* < 0.001). Significant differences were also found between groups in terms of education, with “fence sitters” and “rejecters” having the lowest level of education and “accepters” having the highest; occupational status, with “fence sitters” showing a higher rate of unemployment; financial situations, which had low rate of improvement in the last 3 months for “fence sitters”; and COVID-19 experience, with “accepters” having more direct (10.3 vs. 7.5% of “fence sitters” and 5.8% of “rejecters,” *p* < 0.001) and indirect (79.0 vs. 64.7% of “fence sitters” and 73.2% of “rejecters,” *p* < 0.001) experience with the virus (i.e., had personally been infected or knew someone who contracted the virus). “Fence sitters” had the lowest rate of chronic diseases (17.1 vs. 30.4% in “rejecters” and 32.0% in “accepters,” *p* < 0.001). These sociodemographic and clinical differences accurately reflect the official vaccination policy during the study period, when people who were first offered the vaccine (here divided into “accepters” and “rejecters”) were predominantly older, had chronic diseases, were highly educated (e.g., health workers or teachers), or had priority in the vaccination campaign due to risks of the virus contagion and spread related to their job.

**Table 1:** Sociodemographic, psychological, belief and behavioral differences between “Rejecters,” “Fence sitters” and “Accepters”.

	“Rejecters” (*N* = 138, 6.8%*)	“Fence sitters” (*N* = 575, 28.5%*)	“Accepters” (*N* = 1,302, 64.6%*)	***p*** **-value**	***Post hoc***
**Socio-demographic information**					
Age (years; mean, SD)	49.9 (11.9)	43.1 (11.9)	50.5 (11.8)	**<0.001**	**FS < A/R**
Gender (n, % Male)	69 (50.0%)	272 (47.3%)	645 (49.5%)	0.649	
Education				**<0.001**	
0–8 years (*n*, %)	64 (46.4%)	264 (45.9%)	394 (30.2%)		
9–13 years (*n*, %)	49 (35.5%)	225 (39.1%)	532 (40.9%)		
>13 years (*n*, %)	25 (18.1%)	86 (15.0%)	376 (28.9%)		
Working (*n*, % yes)	81 (58.7%)	285 (49.6%)	713 (54.8%)	0.052	
Being health worker (*n*, % yes)	5 (6.2%)	5 (1.8%)	136 (19.1%)	**<0.001**	
Chronic disease (*n*, % yes)	42 (30.4%)	98 (17.1%)	416 (32.0%)	**<0.001**	
Economic situation in last 3 months				**<0.001**	
Improved (*n*, %)	9 (6.7%)	22 (3.9%)	62 (4.8%)		
Remained the same (*n*, %)	87 (64.4%)	300 (53.5%)	884 (68.6%)		
Worsen (*n*, %)	39 (28.9%)	239 (42.6%)	342 (26.6%)		
**Wellbeing status**				**0.013**	
Good WB (*n*, %)	61 (44.2%)	215 (37.4%)	597 (45.9%)		
Poor WB (*n*, %)	39 (28.3%)	180 (31.3%)	374 (28.7%)		
Depression (*n*, %)	38 (27.5%)	180 (31.3%)	331 (25.4%)		
**COVID-19 experience**					
Personal experience (*n*, % yes)	8 (5.8%) [4.3%]**	43 (7.5%) [23.2%]**	134 (10.3%) [72.5%]**	**<0.001**	
Experience of acquaintances (*n*, % yes)	101 (73.2%) [6.7%]**	372 (64.7%) [24.8%]**	1,029 (79.0%) [68.5%]**	**<0.001**	
**Conspiracy Mentality Questionnaire score** (mean, SD)	25.0 (5.3)	23.7 (4.8)	22.2 (5.5)	**<0.001**	**R>FS>A**
**Protective behaviors** (mean, SD)	−0.4 (1.2)	−0.1 (1.0)	0.1 (0.8)	**<0.001**	**R < FS < A**
**Trust in Media Information sources** (mean, SD)	−0.4 (1.2)	−0.1 (0.9)	0.1 (0.9)	**<0.001**	**R < FS < A**
**Trust in Health Information sources** (mean, SD)	−0.7 (1.2)	−0.3 (0.9)	0.2 (0.9)	**<0.001**	**R < FS < A**
**Frequency use media information sources** (mean, SD)	−0.3 (0.9)	0 (0.8)	0 (0.9)	**<0.001**	**R < FS/A**
**Frequency use Health information sources** (mean, SD)	−0.7 (1.0)	−0.3 (0.9)	0.2 (0.9)	**<0.001**	**R < FS < A**
**Trust in Healthcare Institutions** (mean, sd)	−0.6 (1.2)	−0.3 (0.9)	0.2 (0.9)	**<0.001**	**R < FS < A**
**Agreement with restrictions** (mean, SD)	−0.5 (1.1)	−0.1 (0.8)	0.1 (0.9)	**<0.001**	**R < FS < A**
**COVID-19 Perceived risk** (mean, SD)	−0.3 (1.0)	−0.1 (0.8)	0.1 (0.7)	**<0.001**	**R < FS < A**
**Resilience** (mean, SD)	0.1 (1.1)	−0.1 (0.8)	0 (0.9)	**0.042**	**/**

* *Percentages refer to the total sample included in these analyses (N = 2,015)*.

** *Percentages refer to the total of COVID-19 Personal experience (N = 185) and of acquaintances (N = 1,502)*.

*R, “Rejecters”; FS, “Fence sitters”; A, “Accepters”*.

*Bold values refer to p value < 0.05*.

Psychological and behavioral factors and beliefs were distributed very clearly among groups, as shown in Figure 2: the CMQ scores range from “accepters” (lowest) to “rejecters” (highest), with “fence sitters” in the middle, while protective behaviors, trust and use of media and Health information sources, trust in Healthcare Institutions, agreement with restrictions and COVID-19 perceived risk have the opposite trend: from “rejecters” (lowest values) to “accepters” (highest values). In *post-hoc* comparisons there were no differences between “fence sitters” and “accepters” in terms of frequency use of media information sources.

The findings of the resilience tests are also intriguing, with “rejecters” scoring the highest value, and “accepters” scoring higher than “fence sitters,” who are once again in the most unfavorable position (these differences, however, did not remain in the *post-hoc* comparisons).

The multinomial logistic regression models (Table 2) show that for every additional point of COVID-19 perceived risk, the probability of being a “rejecter” rather than a “fence sitter” was about halved (OR = 0.53, *p* = 0.002). There was also a link between trust and use of the media and health information sources, as well as agreement with restrictions, with each additional point lowering the probability of being a “rejecter” rather than a “fence sitter” by ~30 to 40%. Higher protective behaviors, trust in Healthcare Institutions and agreement with restrictions were also associated to a greater probability of being a “fence sitter” rather than a “rejecter” (OR = 0.76, *p* = 0.049, OR = 0.75, *p* = 0.042 and OR = 0.58, *p* = 0.002, respectively). The CMQ scores was no longer significantly associated with being a “fence sitter” rather than a “rejecter” after covariates adjustment.

**Table 2:** Likelihood of being in the “*Rejecters*” or “*Accepters”* respect to “*Fence sitters”* group: output of the multinomial logistic regression models (one for each factor).

**Rejecters**	OR*	95% C.I.*	***p*** -value**	**Nagelkerke's R^**2**^**
Wellbeing status				0.226
Good WB (*n*, %)	1 (ref)			
Poor WB (*n*, %)	1.17	0.62–2.21	0.653	
Depression (*n*, %)	1.15	0.61–2.18	0.685	
Protective behaviors	0.76	0.59–0.97	**0.049**	0.237
Trust in Media Information sources	0.72	0.55–0.95	**0.040**	0.237
Trust in Health Information sources	0.71	0.55–0.92	**0.024**	0.297
Frequency use media information sources	0.69	0.51–0.94	**0.038**	0.231
Frequency use Health information sources	0.61	0.46–0.81	**0.002**	0.317
Trust in Healthcare Institutions	0.75	0.59–0.97	**0.042**	0.288
Agreement with restrictions	0.58	0.44–0.78	**0.002**	0.255
Conspiracy Mentality Questionnaire	1.02	0.97–1.08	0.415	0.246
COVID-19 Perceived risk	0.53	0.38–0.75	**0.002**	0.240
Resilience	1.17	0.88–1.54	0.333	0.223
**Accepters**	OR*	95% C.I.*	***p*** -value**	**Nagelkerke's R** ^**2**^
Wellbeing status				0.226
Good WB (*n*, %)	1 (ref)			
Poor WB (*n*, %)	0.92	0.63–1.34	0.693	
Depression (*n*, %)	0.73	0.50–1.06	0.120	
Protective behaviors	1.20	1.02–1.41	**0.036**	0.237
Trust in Media Information sources	1.18	1.00–1.39	**0.044**	0.237
Trust in Health Information sources	1.79	1.51–2.13	**0.002**	0.297
Frequency use media information sources	1.08	0.91–1.29	0.389	0.231
Frequency use Health information sources	1.89	1.59–2.26	**0.002**	0.317
Trust in Healthcare Institutions	1.75	1.47–2.08	**0.002**	0.288
Agreement with restrictions	1.27	1.07–1.51	**0.006**	0.255
Conspiracy Mentality Questionnaire	0.94	0.91–0.97	**0.002**	0.246
COVID-19 Perceived risk	1.09	0.89–1.34	0.361	0.240
Resilience	1.00	0.85–1.17	0.954	0.223

* *Adjusted for age, chronic disease, educational level, working status, health-working status, economic situation in last 3 months and COVID-19 experience*.

** *Bootstrap results, based on 500 bootstraps samples*.

*Bold values refer to p value < 0.05*.

**Figure 3:**
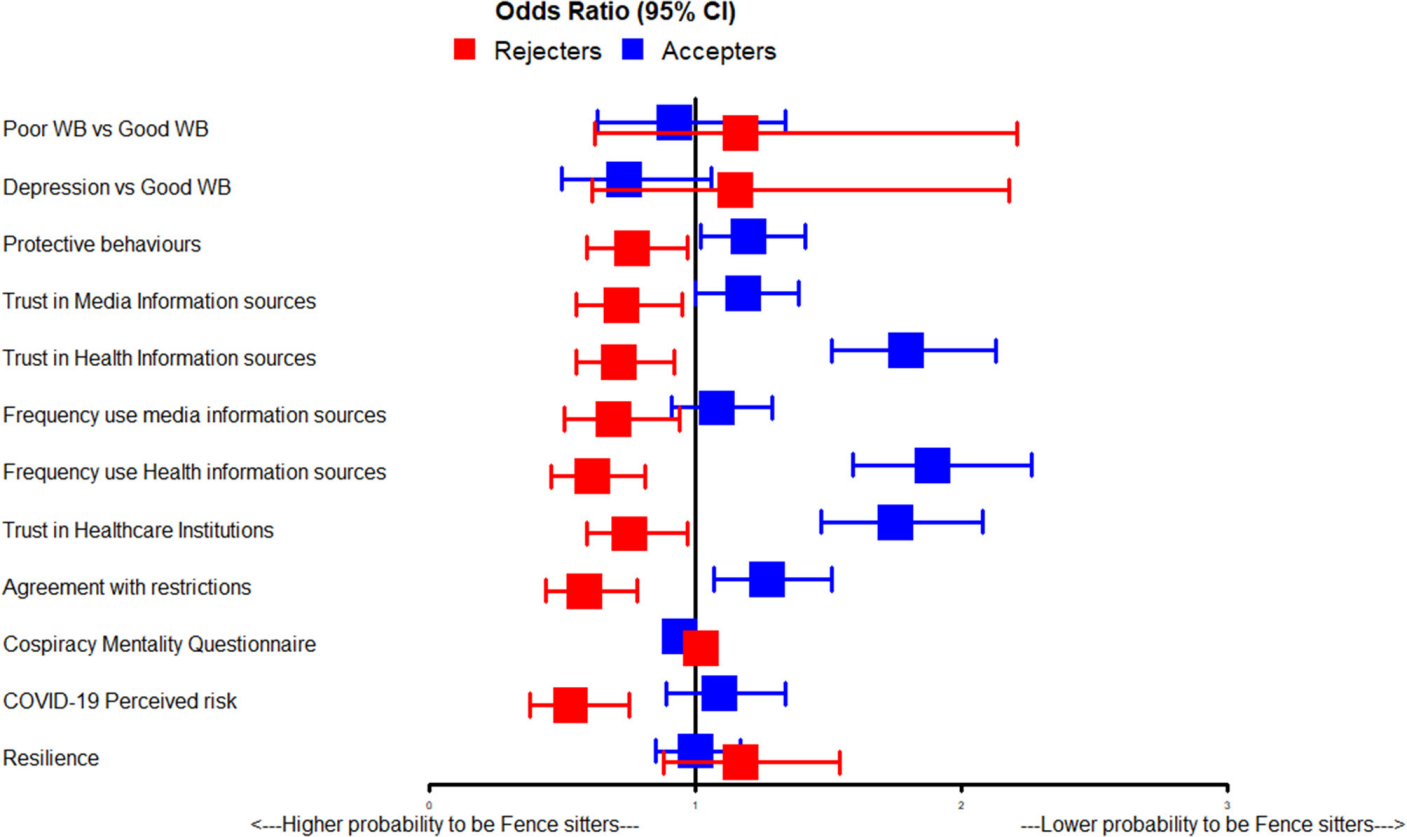
Graphical representation of ORs (and 95%CI) of the multiple logistic regression models.

Increases in specific psychological and behavioral factors were linked to a higher probability of being an “accepter” rather than a “fence sitter.” These factors are: trust in healthcare institutions (OR = 1.75, *p* = 0.002) and trust and frequency of use of health information sources (OR = 1.79, *p* = 0.002 and OR = 1.89, *p* = 0.002, respectively), trust in media information sources (OR = 1.18, *p* = 0.044) and agreement with restrictions (OR = 1.27, *p* = 0.006). The effects of protective behaviors (OR = 1.20, *p* = 0.036) were still significant. On the contrary, a lower Conspiracy Mentality Questionnaire (OR = 0.94, *p* = 0.002) was associated with a higher probability of being an “accepter” rather than a “fence sitter.” After covariates adjustment, COVID-19 perceived risk was no longer significantly associated with being a “fence sitter” rather than an “accepter.” Figure 3 shows an overview of the findings of the multinomial logistic regression models.

## Discussion

Our study found that several factors have been linked to acceptance, fence sitting, or refusal of the COVID-19 vaccine. These include sociodemographic features (such as age, education, economic situation, having a chronic disease, COVID-19 experience), psychological wellbeing, attitudes and beliefs (such as trust in media sources and institutions, trust in institutions, agreement with restrictions, COVID-19 perception risk, conspirative mentality) and behaviors (i.e., protective behavior against the virus, frequency of use of media or institutional informational sources).

### Sociodemographic Factors

Our findings imply that the three identified subgroups have significant differences in some sociodemographic features. Indeed, the majority of “fence sitters” were mainly young people with a low educational level, worsened economic situation in the preceding 3 months, lower rates of both employment as health workers and chronic diseases. No differences between the three groups were found for gender and working status. At odds with this finding, other studies suggested that there is an association between female gender and vaccine hesitancy (12, 16, 18, 19, 22–24). Our results were consistent with previous studies that linked COVID-19 vaccine hesitancy to sociodemographic factors such as younger age (16–21), lower education (16, 18, 30) and lower income (16, 20, 30).

Our results highlight the relevance of education in affecting vaccination behavior and attitudes (i.e., only 15.0% of “fence sitters” had an education level > 13 years, *compared with* 28.9% of the “accepters”). Indeed, we suppose that low education may be linked to poor health literacy, which is related to the ability to obtain, process and understand essential health information and services required to make informed health decisions (42). As a result of this shortcoming, there may be misunderstanding and uncertainty, reducing the willingness to get vaccinated.

Furthermore, we found that economic situations may have a significant impact on the decision to get vaccinated. This may be because individuals who did not experience economic difficulties as a result of the pandemic felt “protected” by the government and were more prone to trust and agree with government policies (i.e., including vaccination campaign).

In addition, we found that “accepters” reported higher rates of both direct and indirect experience with COVID-19 infection than vaccination skeptics; closer interaction with the virus may contribute to a greater risk perception and sensitivity to the need of protecting themselves. However, this point should be further investigated because it contradicts previous results that people who believed they had COVID-19 were less likely to report following lockdown measures (43), and people who had COVID-19 with severe symptoms were more hesitant to take the vaccine than people who did not experience the disease at all (44).

### Psychological Wellbeing

When compared to the “accepters,” the “fence sitters” group reported lower rates of wellbeing status. Individuals with psychological difficulties may vacillate in their decision to get vaccinated due to maladaptive behavior (i.e., reduced medical seeking, lower prevalence of health-protecting behavior, poor self-care and noncompliance with medical prescriptions), which is common among them (45, 46). Individuals with psychological difficulties may be more hesitant to self-protect and follow the vaccination campaign as a result of this predisposition. However, to the best of our knowledge, only a few studies have investigated the relationship between psychological status and COVID-19 vaccination intentions or behavior. Batty et al. (47) discovered that having a pre-pandemic diagnosis of anxiety or depression, or a high score on the distress symptom scale, had no influence on vaccine willingness. Therefore, our findings highlight that “fence sitters” had the highest psychological burden and for these reasons, they require specific attention in light of ongoing vaccination campaigns.

### Attitudes and Beliefs

We observed that trust in both media and health information sources and in healthcare institutions, as well as agreement with restrictions, conspirative mentality and COVID-19 perception risk, were all associated with vaccine behavior or attitudes. Lower levels of trust in media and health information sources and in healthcare institutions, as well as agreement with restrictions, and higher levels of conspiracy mentality, were all linked to a higher likelihood of being in the “fence sitters” group rather than the “accepters” group. Additionally, a higher level of trust in both media and health information sources, as well as in healthcare institutions, agreement with restrictions and an increased COVID-19 risk perception were associated with a higher likelihood of being in the “fence sitters” group rather than the “rejecters” group.

Our results are in line with previous studies indicating an association between COVID-19 vaccine hesitancy and adherence to conspiracy theories (14, 16, 18, 29, 48), poor perception of government measures (20) and a lack of trust/confidence in scientists, healthcare personnel, health institutions and/or the government (12, 16, 20, 22, 28). Furthermore, past research has revealed that conspiracy theories can harm trust in authorities and institutions (49, 50), as well as act as barriers to health protective behavior, including unwillingness to vaccinate (14, 48, 50–53).

We found that “rejecters” had lower COVID-19 perceived risk than “fence sitters” and “fence sitters” had lower COVID-19 perceived risk than “accepters.” Furthermore, increased COVID-19 perceived risk was linked to a higher likelihood of being in the “fence sitters” group rather than the “rejecters” group, even after adjusting for sociodemographic factors. Interestingly, vaccine “accepters” reported the highest levels of COVID-19 perception risk even if their got vaccinated. We may argument that probably this may be a trait-related perception that led them to choose vaccination as protection. Moreover, it is possible that “rejecters” may not have trusted the available information concerning the severity of the COVID-19 virus and hence perceived a low risk. Indeed, earlier research focusing on groups with significant vaccine hesitancy has reported the belief that risks related to the COVID-19 pandemic had been exaggerated by the media and that the pandemic would not last long (25). Indeed, previous studies on vaccine hesitancy (covering both “rejecters” and “fence sitters”) indicated that this group has a low perceived risk (16, 18, 24, 26). Our study may allow a better distinction in risk perception between those who refused and those who were uncertain about their future decision, pointing to a higher perceived risk in those who were unsure about their future decision.

### Behavioral Factors

We found that a higher frequency of using health informational sources, and higher rates of protective behavior were linked to a higher likelihood of being a vaccine “accepter” rather than a “fence sitter.” This finding is consistent with earlier research that identified a link between vaccine hesitancy and either a lesser use of traditional and authoritative information sources (27) or a higher use of media information sources (29, 54). During a global emergency, the frequency with which different information sources, particularly institutional ones, are used is critical. A low rate of usage of institutional information sources may be associated with vaccine reluctance because people are misinformed about vaccines and their efficacy, and they regard them as something out of their control.

We also discovered that “fence sitters” reported COVID-19 associated protective behavior that was lower than to vaccine “accepters” but higher than that of vaccine “rejecters,” which could be related to the trend of risk perception among three groups. We suppose that protective behaviors are closely linked to the risk perception: indeed, an increased risk perceived may be associated with an higher probability that protective behaviors, including vaccination, are implemented.

### Limitations

The length of the survey was the study's principal constraint. Indeed, the COVID-19 vaccine was only offered to specific population groups in Italy in March, April and May 2021 (i.e., healthcare workers, older people, individuals with chronic and disabling diseases and educational staff), as shown by the socio-demographic characteristics of the three groups studied. This limitation may limit the generalizability of these findings to the whole Italian population. To reduce selection bias, we adjusted multinomial logistic regression for all sociodemographic features which were linked to vaccination rates. Therefore, the logistic regression models were adjusted for age, gender, chronic disease, educational level, working (and health-working) status, economic situation in the last 3 months and COVID-19 infection. In this way we were able to manage the potential confounding effect caused by the disparity between the two groups who were offered the vaccination (“accepters” and “rejecters”) and those who were not yet offered the vaccination (“fence sitters”) and were assessed about their willingness to get vaccinated in the future.

Furthermore, in the case of “fence sitters,” we only assessed a snapshot of vaccination views at a single point in time, when vaccination had not yet been proposed to them; thus, we have no way of knowing how vaccine attitudes may evolve in response to circumstantial or individual changes (e.g., COVID-19 spread, economic changes or personal experiences). Finally, the representativeness of the Italian adult population is limited to individuals under the age of 70 who have access to the Internet. Unfortunately, during a pandemic conducting face to face interviews is not recommended since it may favor subjects exposure to the risk of contagion, and for this reason the conduct of an online questionnaire administration was a mandatory choice. The missed involvement of older people and people not acquainted with ITC devices was a necessary limitation to prevent Covid-19 and to promote good health practice.

## Conclusions

The WHO has stated that media messaging about public health issues can have a huge impact on individual behavior. Therefore, the results of this study may be useful in informing governments and addressing specific media communication strategies, particularly for those who are uncertain about getting vaccinated against COVID-19. Specific communication strategies should be developed to improve the frequency of use and trust in health information sources, as well as to alleviate the concerns of vaccine skeptics. The profile of “fence sitters” that emerged from this study is particularly interesting because it highlights a specific profile of a young person, who is poorly educated, has economic difficulties, and is particularly concerned about the pandemic in terms of subjective psychological distress. People in their early 40 s who are poorly educated and have economic difficulties should be the sociodemographic target profile of public programmes aimed at improving vaccine campaign adherence. Given the “fence sitter” group's characteristics, it is likely that this segment of the population is most concerned about the possible side effects of vaccines. From this perspective, targeted information about the vaccinations' potential side effects could persuade a significant number of “fence sitters” to get vaccinated. According to the “five Cs,” to combat vaccine hesitancy (55), communication strategies and public programmes should emphasize the following features: Confidence (i.e., vaccines are important, safe and effective); Complacency (i.e., perception of low risk and disease severity); Convenience (i.e. access issues based on the context, time and specific vaccine being offered); Communications (i.e., decreasing misinformation and infodemic); and Context (i.e., sociodemographic characteristics). To address the public's concerns and build confidence, a true transparent communication is essential.

## Data Availability Statement

The datasets presented in this study can be found in online repositories. Datasets and codes are available here: http://doi.org/10.5281/zenodo.5040719.

## Ethics Statement

The studies involving human participants were reviewed and approved by IRCCS San John of God Fatebenefratelli of Brescia (no. 72-2020). The patients/participants provided their written informed consent to participate in this study.

## Author Contributions

GG: funding acquisition, conceptualization, methodology, original draft, and writing—review and editing. CZ, MZ, CF, and VC: conceptualization, methodology, data curation, data analysis, original draft, writing—review, and editing. Md'A: conceptualization, original draft, and writing—review and editing. GC, FS, MC, TG, LL, and AT: writing—review and editing. All authors contributed to the article and approved the submitted version.

## Funding

This work was supported by Fondazione Cariplo (Grant no. 2020-5195), the Italian Ministry of Health (Ricerca Corrente) and IRCCS Centro San Giovanni di Dio Fatebenefratelli Institutional resources.

## Conflict of Interest

The authors declare that the research was conducted in the absence of any commercial or financial relationships that could be construed as a potential conflict of interest.

## Publisher's Note

All claims expressed in this article are solely those of the authors and do not necessarily represent those of their affiliated organizations, or those of the publisher, the editors and the reviewers. Any product that may be evaluated in this article, or claim that may be made by its manufacturer, is not guaranteed or endorsed by the publisher.
