# Supplementary material for: COVID-19 Vaccine Hesitancy in Italy: Predictors of Acceptance, Fence Sitting and Refusal of the COVID-19 Vaccination
Source: Front Public Health. 2022 Apr 29;10:873098. doi: 10.3389/fpubh.2022.873098 (PMC9098927; doi:10.3389/fpubh.2022.873098)
Supplement: Supplementary file 1 [file Table_1.docx]

**SUPPLEMENTARY MATERIAL**

**Table 1S.**

Items included in each factor

|  | **COVID-19 perceived risk** | **Trust in health institutions** | **Protective behaviours** | **Trust in Media Information sources** | **Trust in Health Information sources** | **Frequency use media information sources** | **Frequency use Health information sources** | **Agreement with restrictions** | **Resilience** |
| --- | --- | --- | --- | --- | --- | --- | --- | --- | --- |
| **N. of Items** | 5 | 4 | 7 | 5 | 6 | 5 | 6 | 5 | 3 |
| ***Cronbach's* α** | 0.707 | 0.893 | 0.791 | 0.894 | 0.931 | 0.843 | 0.904 | 0.835 | .864 |
| **Item 1** | Probability | Family doctor | Hands protections | TV | Health care workers | TV | Health care workers | Thinking that restrictions are too much exceeding (R) | I have a hard time making it through stressful events (R) |
| **Item 2** | Susceptibility | Local Health  Unit | Eyes protections | Newspapers | Health Ministry | Newspapers | Health Ministry | Mask use | It does not take me long to recover from a stressful event |
| **Item 3** | Severity | Health Ministry | Hands disinfectants | Social Network | National Health  Institute (ISS) | Social Network | National Health  Institute (ISS) | Bar/Restaurant limitations | It is hard for me to snap back when something bad happens (R) |
| **Item 4** | Affect-Frightening | National Health Institute (ISS) | Social behaviour | Radio | World Health Organization (WHO) | Radio | World Health Organization (WHO) | Distance learning |  |
| **Item 5** | Affect-Closeness |  | Mask | Influent people | Green number | Influent people | Green number | Curfew |  |
| **Item 6** |  |  | Distance |  | Government website |  | Government website |  |  |
| **Item 7** |  |  | Surface disinfectants |  |  |  |  |  |  |
| **Load. Item 1** | 0.433 | 0.510 | 0.760 | 0.831 | 0.702 | 0.600 | 0.664 | 0.573 | .877 |
| **Load. Item 2** | 0.513 | 0.687 | 0.704 | 0.818 | 0.894 | 0.731 | 0.854 | 0.587 | .469 |
| **Load. Item 3** | 0.450 | 0.877 | 0.754 | 0.625 | 0.894 | 0.606 | 0.885 | 0.824 | .860 |
| **Load. Item 4** | 0.546 | 0.911 | 0.552 | 0.801 | 0.845 | 0.718 | 0.851 | 0.449 |  |
| **Load. Item 5** | 0.430 |  | 0.697 | 0.583 | 0.616 | 0.602 | 0.465 | 0.780 |  |
| **Load. Item 6** |  |  | 0.704 |  | 0.780 |  | 0.706 |  |  |
| **Load. Item 7** |  |  | 0.124 |  |  |  |  |  |  |
| **Percentage of**  **Explained variability** | 0.38 | 0.68 | 0.49 | 0.64 | 0.69 | 0.54 | 0.63 | 0.54 | .69 |

Number of items, internal consistency (Cronbach’s α), items name and their estimated loadings, total deviance explained by the loadings and proportion of variance explained by EFA for each dimension.

**Composition of summary factors:** Factors consist of variables with heterogeneous weights. For example, *Trust in health institutions* is composed almost entirely of Health Ministry and National Health Institute trust, much less determining instead Family doctor and Local Health Unit. *Protective behaviours* consider Surface disinfectants much less than other forms of precaution. As regards *Frequency use of Health information sources*, Health Ministry, National Health Institute and World Health Organization have the greatest weight, while Green number has almost half of it.
